# Antibiotic and Heavy Metal Resistance in Bacteria from Contaminated Agricultural Soil: Insights from a New Zealand Airstrip

**DOI:** 10.3390/antibiotics14020192

**Published:** 2025-02-13

**Authors:** Ali Heydari, Nick D. Kim, Patrick J. Biggs, Jacqui Horswell, Gerty J. H. P. Gielen, Alma Siggins, Collette Bromhead, Juan Carlos Meza-Alvarado, Barry R. Palmer

**Affiliations:** 1 School of Health Sciences, Massey University, Wellington 6021, New Zealand; aliheydari781@gmail.com (A.H.); n.kim@massey.ac.nz (N.D.K.); jacqui.horswell@nzagrc.org.nz (J.H.); c.bromhead@massey.ac.nz (C.B.); jcmeza.alvarado@hotmail.com (J.C.M.-A.); 2 School of Food Technology and Natural Sciences, Massey University, Palmerston North 4410, New Zealand; p.biggs@massey.ac.nz; 3 Infectious Disease Research Centre, Massey University, Palmerston North 4410, New Zealand; 4 School of Veterinary Science, Massey University, Palmerston North 4410, New Zealand; 5 Scion, Rotorua 3010, New Zealand; gjgielen@outlook.com; 6 School of Biological and Chemical Sciences, University of Galway, H91 TK33 Galway, Ireland; alma.siggins@universityofgalway.ie; 7 Ryan Institute, University of Galway, H91 TK33 Galway, Ireland

**Keywords:** heavy metal resistance, bacteria, soil, antimicrobial resistance, PICT

## Abstract

Background/Objectives: Agricultural soils accumulate inorganic contaminants from the application of phosphate fertilisers. An airstrip located at Belmont Regional Park (BRP), near Wellington, New Zealand, has been found to have a gradient of cadmium contamination due to spillage of superphosphate fertiliser. Methods: Soil samples from the BRP airstrip with a gradient of cadmium contamination, were used as a novel source to explore bacterial communities’ resistance to heavy metals (HMs) and any co-selected antibiotic (Ab) resistance. Results: Differences between BRP soil samples with higher levels of HMs compared to those with lower HM concentrations showed significantly more bacterial isolates resistant to both HMs (40.6% versus 63.1% resistant to 0.01 mM CdCl_2_, *p* < 0.05) and Abs (23.4% versus 37.8% resistant to 20 μg/mL tetracycline, *p* < 0.05) in soils with higher initial levels of HMs (1.14 versus 7.20 mg kg^−1^ Cd). Terminal restriction fragment length polymorphism (TRFLP) and 16S rDNA next-generation sequencing profiling investigated changes in HM-induced bacterial communities. Significant differences were observed among the bacterial community structures in the selected BRP soil samples. Conjugative transfer of cadmium resistance from 23–38% of cadmium-resistant isolates to a characterised recipient bacterial strain in vitro suggested many of these genes were carried by mobile genetic elements. Transconjugants were also resistant to zinc, mercury, and Abs. Higher levels of HMs in soil correlated with increased resistance to HMs, Abs, and elevated levels of HMs thus disturbed the bacterial community structure in BRP soil significantly. Conclusions: These findings suggest that HM contamination of agricultural soil can select for Ab resistance in soil bacteria with potential risks to human and animal health.

## 1. Introduction

New Zealand agricultural soils are subject to a range of contaminant inputs, of which inorganic contaminants in phosphate fertilisers and animal remedies are of special interest because they can accumulate over time [1,2]. Fertilisation of agricultural soil with superphosphate can result in the accumulation of heavy metals (HMs), especially cadmium (Cd) [1,2]. Accumulation of HMs in soil can introduce resistance to these HMs (HMR) in soil bacteria and increase the risk of antibiotic resistance (AbR) occurring via cross- or co-resistance [3]. HMs in soil do not degrade [4], but the physicochemical conditions present can determine their mobility and bioavailability [5]. Soil microbiological factors (e.g., bacterial community structure) are also affected by HMs [6]. Contamination of soil by HMs is an on-going issue with severe potential effects on ecosystems and their stability and functions [7]. HM contamination has numerous sources, including agricultural chemicals and fertilisers. The impact of these contaminants may lead to the accumulation of HMs, including Cu, Hg, Pb, Zn, and Cd, in soil with potential risks to human and animal health and selection pressure on soil microbes to develop AbR [3,8]. Previous reports have shown that HMR and AbR can be co-selected by microbes in HM-contaminated environments [9]. This highlights the need to prevent the accumulation of HM contaminants in the biosphere. Sites with gradients of contamination with HMs in soil have been studied for their effects on microbial activity and diversity [10,11], but not to our knowledge with respect to the development of AbR.

We previously reported evidence that contamination with Cd and zinc (Zn) in agricultural soils from the Waikato region of New Zealand, after the addition of superphosphate fertiliser and Zn-containing remedies for animal disease treatment, was associated with selection for HMR and AbR in soil bacteria [12]. Further information was obtained on HM concentration ranges that may select for HMR and AbR from experiments using microcosms with soil supplemented with ranges of concentrations of Cd, Zn, or mercury (Hg) [13]. In the current study, we investigated if selection for HMR and/or AbR was influenced by Cd concentration in the setting of a rural airstrip used for the aerial topdressing of superphosphate fertiliser. The levels of HMR and AbR bacteria along a transect of the airstrip were investigated. Resistant isolates were characterised, and bacterial diversity at a subset of sites was estimated using TRFLP and sequencing techniques. The Belmont Regional Park (BRP) airstrip site is located in rolling hill country in the Wellington region. Loading and take-off operations involve spillage of fertiliser, and the farm airstrip site reflects this history. The airstrip was sampled as a novel site to explore bacterial communities’ resistance to HMs from Group 12 [IIb] of the periodic table and any co-selection for AbR. The gradient of contamination observed at this site provides a novel opportunity to address our aim to study the influence of a range of concentrations of HMs on selection for AbR and associated changes in bacterial community structure in soil at a real-world site; other such studies have usually relied on laboratory-based scenarios [13].

## 2. Results

### 2.1. Belmont Regional Park (BRP) Soil Samples

**Figure 1:**
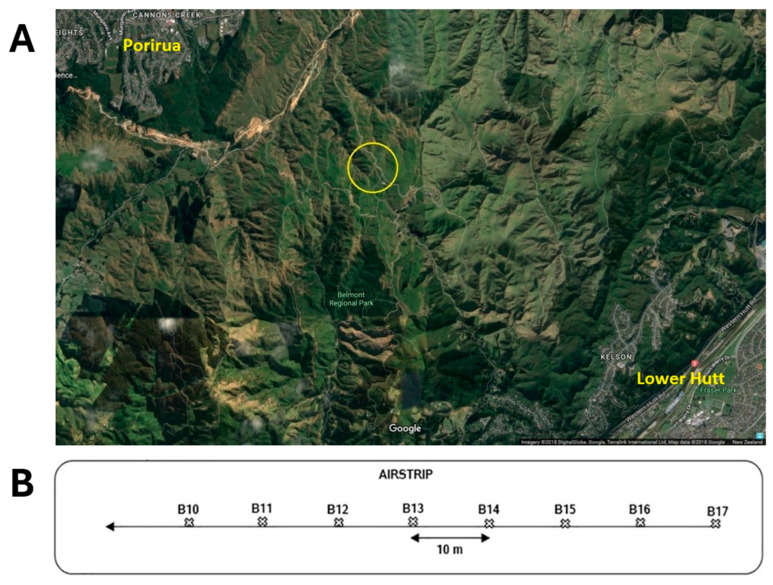
Belmont Regional Park map. (**A**) Airstrip location (yellow circle) Image: Imagery ©2018DigitalGlobe, Google Maps. (**B**) Sampling scheme at Belmont Regional Park airstrip.

At BRP, eight sites (labelled B10–B17) were sampled at 10 m intervals along a transect running the length of a farm airstrip used for the aerial topdressing of superphosphate fertiliser. The soil samples were categorised as clay loam soils with granular structure [14]. The Cd concentration showed a sharp increase from down-hill subsite B10 (assumed to be closest in soil nutrient/mineral composition to the BRP farm paddocks “background” state) to the top-hill subsite B17, which was closest to the fertiliser storage bunker (Figure 1).

HM concentrations and other physicochemical properties of the soil at each subsite are detailed in Table 1. Subsite B17 had the highest Cd and Zn concentrations, B14 had intermediate levels, while B10 contained the lowest content of these elements.

### 2.2. Plate Counts from Subsites’ Soil Samples

**Figure 2:**
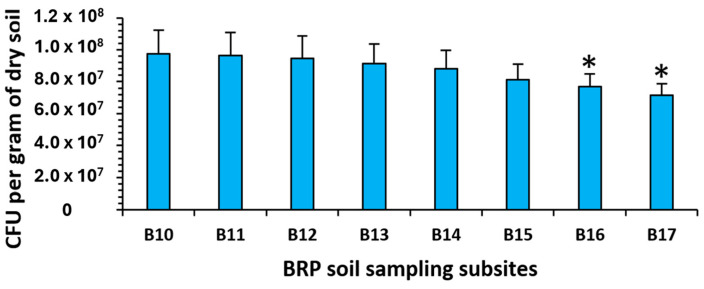
Total plate counts of bacteria (per gram of dry soil) from Belmont Regional Park subsites soil samples (* *p* < 0.05 compared to B10 soil bacteria total CFU).

Bacterial plate counts on R2A agar for soil samples from the BRP subsites showed a steady decrease in bacterial numbers as subsites neared the start of the airstrip (B17). Significant differences (*p* < 0.05) between total bacterial colony-forming units (CFUs) were observed for subsites B16 and B17 (high HM content) when compared to subsite B10 (lowest HM) (Figure 2).

### 2.3. HM Resistant Bacteria from Subsite’s Soil Samples

**Figure 3:**
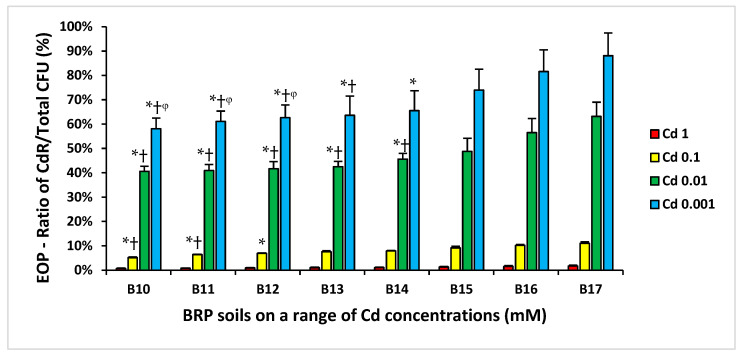
Mean ratios of CdR/total bacterial CFUs, over a range of Cd concentrations, for BRP subsites soil samples. * *p* < 0.05 compared to B17 soil bacteria CFU ratio selected on the same Cd concentration; † *p* < 0.05 compared to B16 soil bacteria CFU ratio selected on the same Cd concentration; ᵠ *p* < 0.05 compared to B15 soil bacteria CFU ratio selected on the same Cd concentration. B10–B17 identify the subsites where soil samples were taken.

Bacteria from soil samples from BRP were cultured on R2A agar supplemented with a range of concentrations of Cd, Zn, and Hg, and efficiency of plating (EOP) ratios (HMR CFUs/total CFUs) were calculated. A three-way ANOVA analysis was used to compare the EOPs from each subsite, with distance from the beginning of the airstrip and HM concentrations in soil samples as independent variables. Soil moisture content, pH, total C and N content, and Olsen *p*-values were covariates in the analysis. ANOVA analysis for these bacterial CFU ratios (Figure 3) showed very similar trends, where soils with higher HM concentrations (B17 and B16) had significantly higher EOPs for HMR compared to the subsites with lower HM concentrations (B11 and B10). In addition, there were significantly higher EOPs when the soils were plated onto agar plates containing lower concentrations of HMs (*p* < 0.05) (Figure 3, Supplementary Figures S1 and S2).

The soil samples with higher concentrations of HM had higher EOPs for Cd resistance (CdR) compared to the samples with lower concentrations of HMs. Furthermore, Cd 0.1 mM-resistant/total bacterial CFU ratios of B11–B10 subsites were lower when compared to those from B17–B16 subsites. Cd 0.01 mM and Cd 0.001 mM CdR/total bacterial CFU ratios of B10 to B14 subsites were lower when compared to those from B16 and B17 subsites (Figure 3). A similar pattern of resistance to Zn and Hg was also observed (Supplementary Figures S1 and S2).

### 2.4. Antibiotic Resistance in Subsites’ Soil Samples

**Figure 4:**
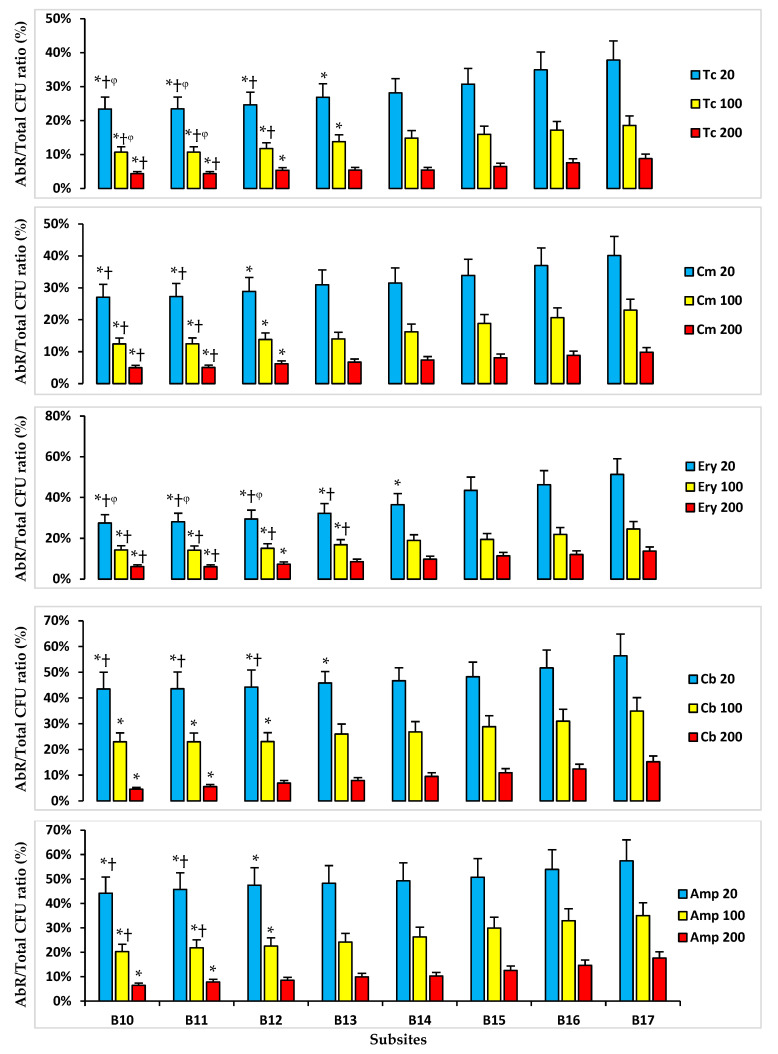
Mean ratios of AbR/total bacterial CFUs, selected on a range of antibiotic concentrations, for BRP subsites soil samples. * *p* < 0.05 compared to B17 soil bacteria CFU ratio selected on the same Ab concentration; † *p* < 0.05 compared to B16 soil bacteria CFU ratio selected on the same Ab concentration; ᵠ *p* < 0.05 compared to B15 soil bacteria CFU ratio selected on the same Ab concentration. B10–B17 identify the subsites where soil samples were taken.

Examination of bacterial resistance to a range of antibiotics (tetracycline (Tc), chloramphenicol (Cm), erythromycin (Ery), carbenicillin (Cb), and ampicillin (Amp)) from different classes was performed for subsites’ soil samples, and the results were analysed by three-way ANOVA (Figure 4). The soil samples with higher levels of HMs (B17 and B16) had significantly higher AbR/total bacterial CFU ratios compared to the soils with lower HM concentrations for all investigated antibiotics (sites B11 and B10).

### 2.5. Characterisation of Cd Resistance Genes

Forty independent CdR isolates from each BRP subsite (n = 320) were selected, and DNA was isolated from each to test for the CdR genes *cadA* and *czcA* by PCR. Amplification of *czcA* was common in these isolates, and the highest levels of CdR gene-positive isolates were found in subsites B17 to B15 (>80%). The lowest occurrence of CdR gene-positive isolates (20%) was found at subsites B10 and B11. Meanwhile, isolates carrying *cadA* were predominantly found at subsites B17–B15 (Table 2). Of the 320 CdR bacterial isolates screened, 175 (54.7%) carried *czcA* and 5 (1.6%) carried *cadA*. Two *cadA* positive isolates and 50 carrying czcA were capable of transferring CdR to the recipient *Pseudomonas aeruginosa* strain MUW001 (Table 2).

Broth microdilution (BM) analysis to determine MIC values for Tc, Cm, Ery, Cb and Amp (according to EUCAST ECOFF recommendations) was performed for the transconjugants of MUW001 that had obtained *czcA* and *cadA*. Overall, more isolates with *czcA* were resistant to Tc (75%) and Cm (74%) than isolates that received *cadA* (Tc: 59%, Cm: 61%). Of the isolates that obtained either the *czcA* or *cadA* gene, 82% to 88% were resistant to Ery, Cb, and Amp.

All transconjugants that had received *czcA* or *cadA* were resistant to 0.1 and 1 mM Cd. In addition, 100% of *czcA^+^* transconjugants showed resistance to 1 and 5 mM Zn. For the transconjugants that received *cadA*, there was 100% resistance to 1 mM and 97% to 5 mM of Zn. Also, 78% of transconjugants carrying the *czcA* gene could grow on 0.1 mM Hg, while 96% of them grew on 0.01 mM of Hg, while 31% of the *cadA^+^* transconjugants were resistant to 0.1 mM Hg, and 73% grew on 0.01 mM of Hg.

### 2.6. Pollution Induced Community Tolerance (PICT) Assay

**Figure 5:**
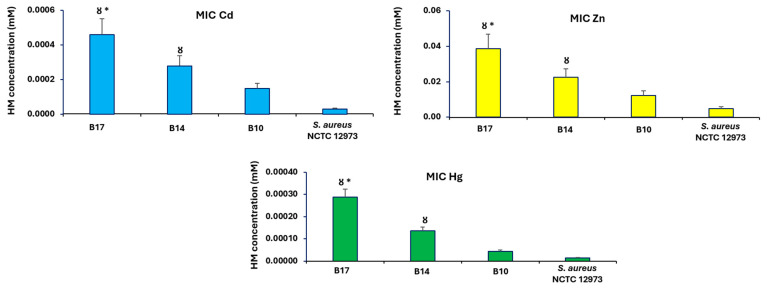
Mean MIC values from PICT assay using HMs (Cd, Zn or Hg) for bacteria from BRP sites with high (B17), intermediate (B14) and low (B10) Cd content. Ȣ *p* < 0.05 compared to HM MIC values for bacteria from B10 soil; * *p* < 0.05 compared to HM MIC value for bacteria from B14 soil. B10, B14, and B17 identify the subsites where soil samples were taken. *S. aureus* NCTC 12973 was used as a negative control.

Bacteria isolated from three subsites of BRP (B17, B14, and B10) were subjected to further analysis given they respectively had the highest (B17), intermediate (B14), and lowest (B10) concentrations of Cd and Zn amongst all BRP subsites soil samples. PICT analysis of HM MICs showed that the MICs for Cd, Zn, and Hg for bacterial consortia from BRP subsites B17 and B14 were significantly higher (*p* < 0.05) than the consortium from B10 soil. The MICs for Cd, Zn, and Hg for B17 soil bacteria were higher when compared to bacteria from B14 soil (Figure 5).

**Figure 6:**
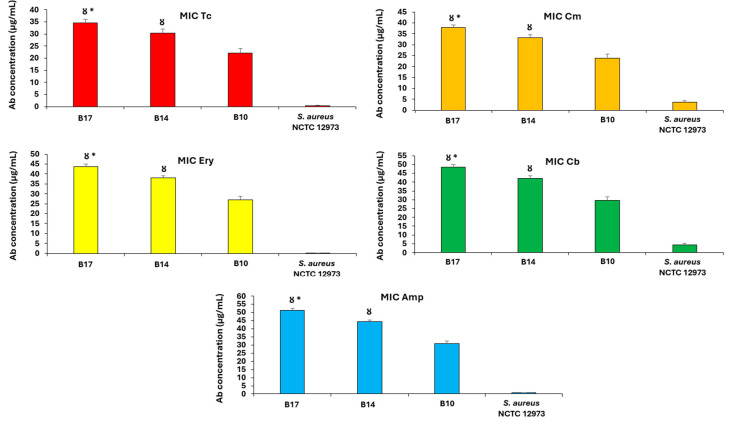
Mean MIC of PICT assays for antibiotics (Tc, Cm, Ery, Cb, Amp) for consortia from BRP sites with high (B17), intermediate (B14), and low (B10) HM content. ^Ȣ^ *p* < 0.05 compared to Ab MIC values for bacteria from B10 soil; * *p* < 0.05 compared to Ab MIC value for bacteria from B14 soil. B10, B14, and B17 identify the subsites where soil samples were taken. *S. aureus* NCTC 12973 was used as a negative control.

Three BRP subsites (B17, B14, and B10) underwent PICT analysis to determine their mean MIC values for five antibiotics (Tc, Cm, Ery, Cb, and Amp). Overall, bacterial consortia from BRP subsites B17 and B14 had higher (*p* < 0.05) MICs for all Abs when compared to the consortium from B10 soil. B17 soil bacteria Ab MIC were also higher when compared to bacteria from B14 soil (Figure 6).

### 2.7. Broth Microdilution (BM) Assays

**Figure 7:**
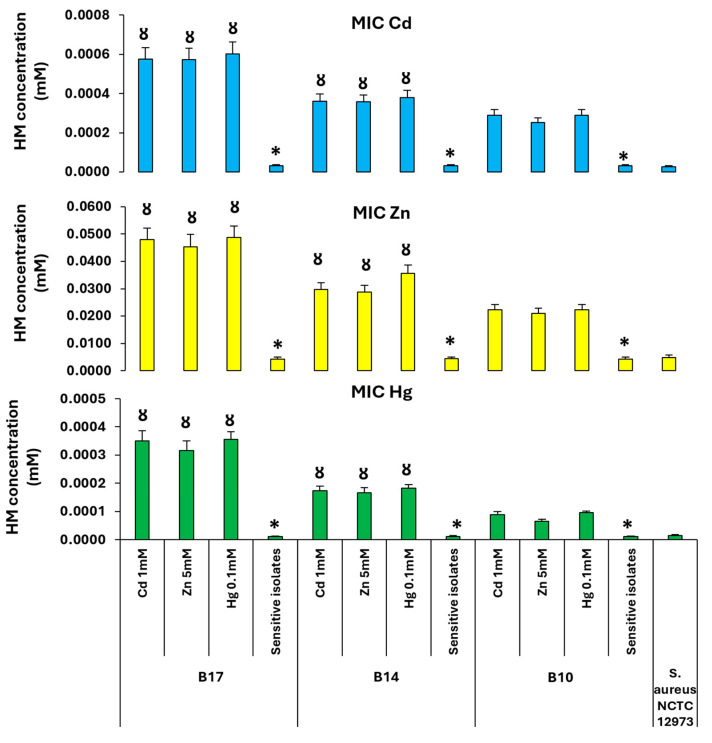
Mean MIC values of BM assay with Cd, Zn, and Hg for the HMR isolates from BRP sites with high (B17), intermediate (B14), and low (B10) HM content. * *p < 0.05* compared to HM MIC value for HMR bacteria from the same soil; Ȣ *p < 0.05* compared to HM MIC value for the bacteria from B10 soil and selected on the same HM concentration. B10, B14, and B17 identify the subsites where soil samples were taken. *S. aureus* NCTC 12973 was used as a negative control.

MIC for Cd, Zn, and Hg was determined by BM assay on isolates that had HMR based on growth on media with high concentrations of HMs (Cd 1 mM, Zn 5 mM, or Hg 0.1 mM). HM-resistant isolates for the three BRP soil subsites that had high (B17), intermediate (B14), and low (B10) concentrations of HMs (Cd and Zn) were compared. It was observed that all HM-resistant isolates from sites B17 and B14 had higher MICs when compared to the B10 isolates. All HM-sensitive bacteria from all sites had lower MIC when compared to HM-resistant isolates from the same soil subsite (Figure 7).

**Figure 8:**
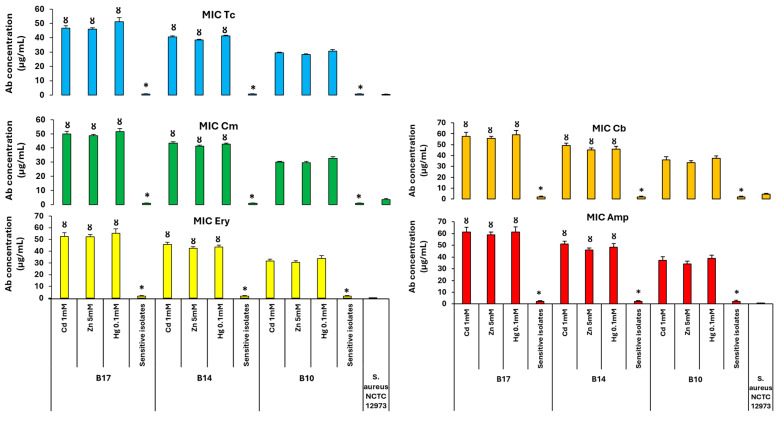
Mean MIC values of BM assay with Tc, Cm, Ery, Cb, and Amp for the HMR isolates from BRP sites with high (B17), intermediate (B14), and low (B10) HM content. * *p < 0.05* compared to Ab MIC value for HMR isolates from the same soil; Ȣ *p < 0.05* compared to Ab MIC value for the bacteria from B10 soil and selected on the same HM concentration. B10, B14, and B17 identify the subsites where soil samples were taken. *S. aureus* NCTC 12973 was used as a negative control.

MICs for Tc, Cm, Ery, Cb, and Amp were determined by BM assay on isolates that had HMR based on growth on media with high concentrations of HMs (Cd 1 mM, Zn 5 mM, or Hg 0.1 mM). HMR isolates were compared for the three BRP soil subsites that had high (B17), intermediate (B14), and low (B10) concentrations of Cd and Zn. It was observed that all HMR isolates from sites B17 and B14 had higher Ab MIC for all antibiotics when compared to the B10 isolates. All HM-sensitive bacteria from all sites presented lower MICs for all Abs when compared to HM-resistant bacteria from the same soil subsite (Figure 8).

### 2.8. Terminal Restriction Fragment Length Polymorphism (TRFLP) Analysis of Soil DNA from BRP Subsites

TRFLP analysis of BRP soil samples’ bacterial communities showed there was >80% similarity between the bacterial communities from the B17, B16, and B15 subsites. Additionally, >80% similarity was observed between bacterial communities from B10 to B14 subsite samples. Between all the bacterial communities from BRP soils, there was >40% similarity (Supplementary Figure S3). Three-way ANOVA showed there were significant differences between the relative abundance of terminal restriction fragments in B17, B16, and B15 subsites’ bacterial communities compared to the remaining BRP soil samples (*p* < 0.05) (Supplementary Figure S3).

### 2.9. 16S rDNA Analysis of Microbial Community Structure

**Figure 9:**
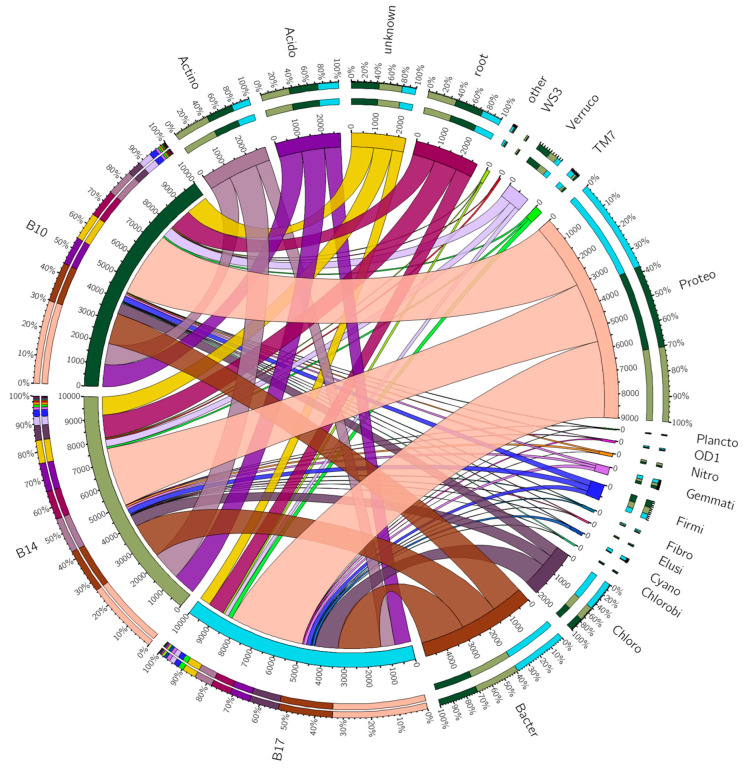
A chord diagram showing the relationship between the top 20 phyla categories by abundance for the three subsites B10, B14 and B17, using the taxonomy results from QIIME2. This resulted in 17 phyla, and the categories “root”, “unknown” and “other”. These refer to sequences that map to the bacterial kingdom, and those that are from unknown phyla, and all remaining phyla aggregated, respectively. All relative abundances have been scaled to 10,000 units for the purposes of plotting and visualisation. The three sites are on the left-hand side, and the phyla are on the right-hand side. Thirteen phyla are abbreviated (Acido: Acidobacteria; Actino: Actinobacteria; Bacter: Bacteroidetes; Chloro: Chloroflexi; Cyano: Cyanobacteria; Elusi: Elusimicrobia; Fibro: Fibrobacteres; Firmi: Firmicutes; Gemmati: Gemmatimonadetes; Nitro: Nitrospirae; Plancto: Planctomycetes; Proteo: Proteobacteria and Verruco: Verrucomicrobia), whereas four (Chlorobi, OD1, TM7, and WS3) are not.

**Figure 10:**
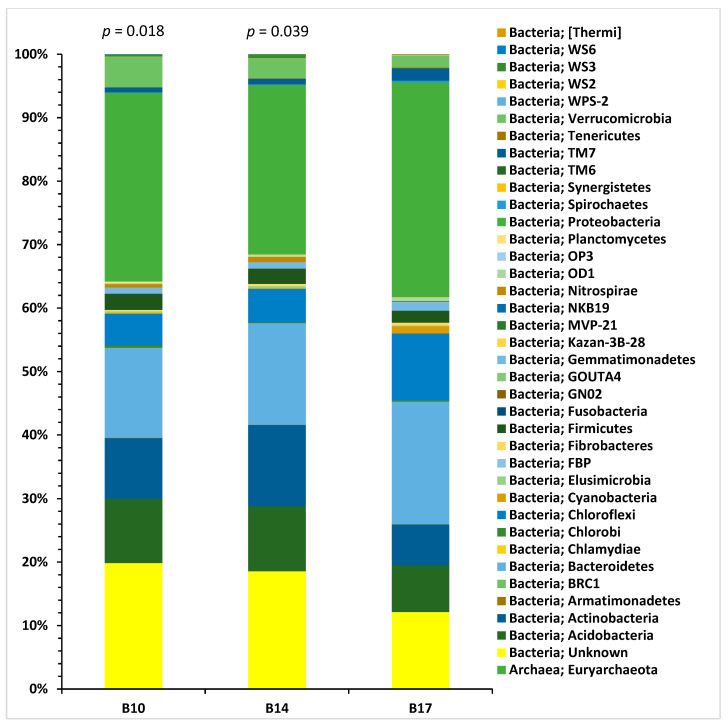
Assignment of sequenced metagenomic next-generation 16S rDNA gene to various bacterial phyla from BRP sites with high (B17), intermediate (B14), and low (B10) HM content. *p*-values compared to the B17 sample.

Analysis of metagenomic 16S rDNA sequence data of bacterial communities from specific BRP soil samples (B10, B14, and B17) was performed with QIIME software, v.2 [8]. B10 (70 m from the start of the airstrip) and B14 (30 m from the start of the airstrip) soil samples were compared to the B17 (at the start of the airstrip) subsite. Due to the number of taxonomic levels detected, a chord diagram summarising this data is only shown for the phylum level (Figure 9). There were significant differences between the number of operational taxonomic units (*p* < 0.05) at each subsite (Figure 10). The most abundant phyla detected, in descending order, were *Proteobacteria*, *Bacteroidetes*, *Actinobacteria*, *Acidobacteria,* and *Chloroflexi*. The phyla *Acidobacteria* and *Chloroflexi* differed the most in abundance between the subsite samples (*p* < 0.05). Supplementary Table S1 lists the *p*-values for comparisons of the number of 16S rDNA gene reads in B10 and B14 compared to B17 for the taxonomical levels including phyla, class, order, family, genus, and species. The alpha diversity at the three subsites is shown in Table 3. It was greatest by most metrics at subsite B10, compared to subsites B14 and B17.

### 2.10. Bacterial Isolates Identification Using 16S rDNA Sequencing

16S rDNA amplimers were amplified by PCR from DNA samples from n = 30 individual bacterial isolates that had successfully mobilised CdR in conjugation trials. The 16S rDNA amplicons were sequenced, and these outputs were compared to the NCBI Bacterial Database using the blastn suite to identify the isolates. Supplementary Table S2 lists the identities of these isolates, including their soil sample origins, descriptions from NCBI, and the percentage identity to the candidates on the NCBI database. Most of the isolates that successfully transferred CdR genes to recipients were from the genera *Pseudomonas, Achromobacter, Stenotrophomonas* (Pseudomonadota phylum), and *Chryseobacterium* (Bacteroidota phylum).

## 3. Discussion

The presence of elevated levels of HMs in soil can change many phenotypic characteristics of bacteria in soil, for example, production of extracellular compounds involved in biofilm production, tolerance features in soil bacteria to HMs and altered metabolism of bacterial cells. This study observed a concentration gradient of superphosphate-related contaminants with the maximum concentration observed nearest to an airstrip loading area (subsite B17) and the lowest occurring at the end of the runway (subsite B10). The actual concentration that influences the various microorganisms in the soil is determined by the bioavailability of the contaminants. Factors that may influence bioavailability include soil fertility and organic matter. Therefore, bioavailability is partly determined by interactions between the living organism and its soil environment, which in this study may have contributed to the level of HM and Ab resistance.

Overall, BRP subsite bacterial CFUs were higher in sites with lower HM levels compared to the subsites with higher levels of HMs (Figure 2). Additionally, the proportions of HMR and AbR bacteria from the subsites with higher HM levels (B17) were greater than those from subsites with lower HM levels (Figure 3 and Figure 4). It has been reported that bacteria can develop mechanisms to manage metal ion uptake through metalloproteins and other pathways normally reserved for essential organic or inorganic ions [15,16]. High levels of HM accumulation in B17 soils could result in a higher HMR bacterial population, particularly in soils with HM levels higher than a bacteria’s tolerance threshold [17,18]. Moreover, HMR is more likely to occur in soils with elevated levels of non-essential HMs lacking biological functions (e.g., Cd and Hg). The higher extractable and bioavailable HMs may contribute conjointly to HMR and AbR, considering resistance mechanisms may occur through similar cellular efflux pumps as well as resistance genes being located on the same genetic elements [3,19,20,21]. At the BRP site, gradients for elements, including phosphorus and fluorine, are also present due to the contamination by superphosphate, and it is certainly possible these gradients affect selection for both HM and Ab resistance.

HGT through mobile genetic elements among soil bacteria is the most important pathway leading to the acquisition of HMR and AbR [22,23]. Previous reports identified that *cadA* and *czcA* are located on plasmids and transposons, which suggests these genes are spread across bacterial species by HGT [24,25,26,27]. We observed *czcA* was more frequent in isolates from all soil sample sites compared to *cadA*. Furthermore, we observed HGT of *czcA* occurred for 28.6% of isolates carrying it. In contrast, we noticed lower occurrence and less mobilisation for *cadA*. The *czcA* gene encodes a transmembrane helical domain (TMH IV) of efflux-RND proteins engaged in Zn^2+,^ Co^2+^, and Cd^2+^ efflux [28]. The *cadA* gene encodes a Cd^2+^-ATPase protein transporter that can confer Zn resistance [24,26,29,30]. There is evidence that an increase in these genes’ presence may be due to HM contamination. For instance, Oger et. al. (2001) reported that there was a significant increase in the occurrence of *cadA* in bacterial communities in soils with elevated levels of HMs [24]. Similarly, it has been suggested that the occurrence of *czcA* in soil bacteria can be selected by HM contamination pressure [26,31]. This agrees with the trends we observed for CdR gene presence and gene mobility. *czcA* may have been more prevalent in our samples due to the profile of the bacterial community in the soil; for example, *czcA* has been found to be common in *Pseudomonas* spp. in a study of Cd-contaminated activated sludge [32]. The greater prevalence of the *czcA* gene may mean it has a more important role than *cadA* in the spread and persistence of CdR, but as these phenomena are influenced by many factors, it is difficult to be explicit about this.

To investigate the impact of HM soil content, HMR and AbR profiles of sites with low (B10), intermediate (B14), and high (B17) HM content were examined by plate culturing for HMR and BM analysis for AbR with the resistance status of the isolates to antimicrobials designated according to the defined Ab resistance breakpoint concentration (20 µg mL^−1^) [33,34]. The PICT assays revealed higher MICs for HMs and Abs for BRP bacterial communities from the high and intermediate HM content soils compared to the low HM content soils, agreeing with previous studies in pastoral soils [35,36,37]. Additionally, reports indicate higher bacterial AbR is associated with higher levels of HMR [36,38]. This co-resistance for Ab can occur in bacterial isolates with different levels of HmR [35]. It is possible that high phosphorous and/or organic carbon levels observed in the high HM soil can lead to more bioavailability of the HMs involved in bacterial resistance development [36,39]. As such, higher levels of Cd and Zn in pastoral soil, due to higher levels of P, may explain the subsequent co-selection of Abs resistance in the presence of the HMs [36,39]. Together these culture-based and genetic findings suggest that contamination of agricultural soil with HMs can select for elevated prevalence of mobile resistance elements with potential to participate in the evolution of multi-resistant bacterial pathogens that may be a significant risk to human and animal health, conclusions supported by our other reports on this phenomenon [12,13]. Practical measures for mitigating the risks of resistance proliferation in agricultural settings might include reducing the use of mineral fertilisers and greater reliance on regenerative agriculture techniques to reduce further HM contamination and enhance the bioavailability of existing soil nutrients and trace elements.

The soil samples collected from BRP pastoral subsites were subjected to TRFLP analysis to explore associations between HM profile and variations in bacterial terminal restriction fragments (T-RFs). TRFLP analysis of bacterial 16S rDNA gene profiles is considered a robust tool to analyse microbial diversity in soils from similar land, which allows the comparison of microbial community structures [40,41,42]. TRFLP analysis of the BRP soil samples showed that subsites with high HM content (B17 and B16) had more variation in bacterial T-RFs abundance compared to other BRP subsites soil samples (Supplementary Figure S3). Some reports indicate that HMs present in soil impose changes on bacterial community structures, and these changes affect almost all T-RF abundances from bacterial communities [43,44,45]. The TRFLP analysis showed that higher HM content resulted in greater changes to soil bacteria community structures than where lower HM concentrations occurred. This agrees with a report of the existence of fundamental relationships between the HM levels and the evolution of bacterial community structures in soil [46].

In the current study, employing 16S rDNA sequencing analysis revealed that bacterial community compositions in the BRP soil samples decreased in diversity as HM levels increased, potentially from the selection pressure of HM contaminants. *Acidobacteria* and *Chloroflexi* differed most in relative abundance of the major phyla in sites with low and intermediate HM content (sites B10 and B14, respectively) in contrast with a site with high HM content (B17). The most prevalent bacterial phylum in BRP soil samples was Proteobacteria, which made up 76.7% of the isolates identified using 16S rDNA sequencing (including the genera *Pseudomonas*, *Achromobacter,* and *Stenotrophomonas*). Other phyla of note were Bacteroidetes (10% of isolates), Actinobacteria (6.7% of isolates), Acidobacteria (8% of isolates), and Chloroflexi (3.5% of isolates). Reports of phylogenetic studies of soil samples report predominant bacteria belong to the phyla of Proteobacteria, Actinobacteria, and Acidobacteria [47,48,49,50]. Various studies have also shown that these phyla are also highly likely to be carriers of *czcA* and *cadA* genes. For example, bacteria carrying *czcA* are usually from the phyla Proteobacteria (e.g., the genera *Burkholderia, Pseudomonas, Ralstonia, Cupriavidus,* and *Shewanella*), Actinobacteria, Verrucomicrobia, and Bacteroidetes (e.g., *Chryseobacterium*) [27,31,51]. Other groups analysing Cd-resistant bacteria by 16S rDNA sequencing determined these bacteria were members of the Proteobacteria, Bacteroidetes, and Actinobacteria phyla and the genera *Chryseobacterium*, *Cupriavidus*, *Curtobacterium*, and *Sphingomonas* [52]. Overall, our findings outlining bacterial diversity being reduced where HM levels were highest agree with these previous observations on soil samples.

Limitations of the study include that we concentrated on a single site (the BRP airstrip); further studies at other airstrips used for superphosphate topdressing could validate our findings. Sampling was restricted to a single time point; it is unknown if the findings would remain stable over time and are affected by factors such as soil moisture levels or ambient temperature. Resource constraints meant that only limited genomic data could be generated from selected DNA samples; more exhaustive genomic analyses could add detail to our findings, such as if the prevalence and diversity of antibiotic resistance genes at the subsites were associated with Cd levels. We restricted our culture-based investigations to conditions that favoured the growth of heterotrophic bacteria; using different culture conditions could extend these findings to include what is happening with fungi and other bacteria.

## 4. Materials and Methods

### 4.1. Belmont Regional Park (BRP) Site

Soil sampling from the BRP (GPS Coordinates −41°11′24.00” S and 174°52′30.00” E), close to Wellington, New Zealand, was performed at a pastoral livestock farm with granular soil and a fertiliser storage shed adjacent to an airstrip. Sampling was performed in December 2016 from eight subsites, 10 m apart, starting from the end of the airstrip (subsite B10, Figure 1). Subsite samples (n = 8) of the upper soil horizon (0–10 cm) were collected in a straight line transect at each subsite using a 10 cm depth foot corer. The length of the transect was determined from previous sampling, which showed that the main contaminant gradient extended to 70 m down the runway in the direction the loaded aircraft travelled during take-off. The choice of 10 m intervals between subsites provided suitable granularity of both the gradient and real changes between each sampling point. Sampling was performed from the least to the most contaminated subsites; the sampling locations are illustrated in Figure 1. At each of the eight locations (B10–B17), five 10 cm deep soil plugs were collected across a 1 m^2^ area (in a “5-dice” pattern) and combined to make a single soil composite representative of that area. Composite samples were transferred under chain of custody to Hill Laboratories, Hamilton, NZ (accredited by International Accreditation New Zealand) for the analytical chemistry testing, which is seen as suitable (and is routinely used for) regulatory testing purposes. Each composite sample was tested for pH, dry matter, total organic carbon, Olsen and total P, and 33 other trace elements. Results for Cd, Hg, Zn, Fe, and P (Table 1) are based on an accredited method (USEPA 200.2) involving nitric/hydrochloric acid digestion of the < 2 mm fraction, with trace-level analysis by ICP-MS. Within this context, the trace element results for repeated analysis of any given mixed soil sample are typically reproducible to under ±5% of the reported value.

### 4.2. Physicochemical Features of Soil Samples

The BRP subsites soil structure and chemical profile information was assessed by Hill Laboratories Ltd. (Hamilton, New Zealand). Soil samples from BRP were kept up to 12 h in the dark within air impermeable bags at 4 °C until the commencement of laboratory studies. Soil preparation before physicochemical analysis included overnight airdrying at 35–40 °C until 4% residual moisture was achieved. The pH of the soil was measured by potentiometric determination on a soil/water slurry 1:2 (*v/v*). Phosphorous was determined by Olsen extraction followed by molybdenum blue colorimetry. Trace levels of 33 metals were determined on dry samples by nitric/hydrochloric acid digestion and inductively coupled plasma mass spectrometry (ICP-MS), keeping fractions <2 mm. Fluoride content was measured by an ion-selective electrode, while organic carbon was determined by catalytic combustion (900 °C O_2_) separation following removal of carbonates by acid pre-treatment.

### 4.3. Heavy Metal (HM) and Antibiotic (Ab) Stock Preparations

The preparation of stock solutions of metal salts was as described previously [12] and used as additives to amend media to culture soil bacteria. The high water solubility of CdCl_2_, ZnSO_4_∙7H_2_O and HgCl_2_ made them suitable for culture-based experiments [53,54,55,56,57].

Five different antibiotics (Abs) belonging to the tetracyclines, chloramphenicol, antipseudomonal penicillins, macrolides, and penicillin groups were used to assess antimicrobial resistance. These were tetracycline (Tc), chloramphenicol (Cm), carbenicillin (Cb), erythromycin (Ery), and ampicillin (Amp), respectively. Initial stock concentrations of 0.2 g mL^−1^ of these Abs were prepared in dimethyl sulfoxide (DMSO). The Abs stocks were sterilised by passing them through sterile 0.45 µm syringe filters and stored at −20 °C [58,59].

### 4.4. Media Preparation

Reasoner’s 2A (R2A) Agar was used as a general solid culture media and amended with Cd, Zn, or Hg salts or Abs as described elsewhere [12]. The growth of fungi and yeasts was inhibited with 100 µg mL^−1^ of filter sterilised cycloheximide in DMSO added to the basal, HM- and Ab-supplemented media before pouring the plates.

### 4.5. Bacteria Culturing (Extraction, Serial Dilution and Plating)

Large soil particles and ground fragments (stones, plant debris) were removed by sieving samples through a 5 mm aperture. Following overnight airdrying at 35–40 °C, 10 g of soil dry weight were added to 90 mL of sterile 1× phosphate-buffered saline (PBS) buffer (pH = 7.0). The mixture was shaken at 200 rpm at 4 °C for 1 h using a refrigerated shaker incubator. Six universal bottles containing 9 mL of sterile 1× PBS buffer were allocated to each soil sample to make serial 10-fold dilutions (10^−2^ to 10^−8^). Aliquots (100 µL) of each serial dilution were spread on plates containing basal, HM- or Ab-supplemented media in triplicate. Plates were incubated at 25 °C for 14 days. Following the incubation, total colony-forming units (CFUs) of plates containing 10–300 colonies were counted. The mean values from each set of triplicates and total CFU obtained per g of soil dry mass were calculated as previously described [12].

### 4.6. Pollution Induced Community Tolerance Assay

The tolerance of a community of microbes to a range of antimicrobial agents at various concentrations was determined by a microtiter plate culturing-dependent method called pollution-induced community tolerance (PICT). To achieve an inoculum at the desired density, optical density (OD) was measured spectrophotometrically using a McFarland 0.5 turbidity standard. To reach the desired number of cells in liquid culture (5 × 10^5^ mL^−1^), suitable dilutions in R2A medium were performed. The assay was done on 96-well microplate plates, each containing a 100 µL aliquot of extracted bacterial dilution (5 × 10^5^ bacterial cell/mL), 99 µL of 2× R2A broth, and 1 µL of each HM or Ab concentration. Each treatment condition had three technical replicates, and the plates included negative (basal R2A) and positive (excess HM or Ab) controls. OD adjustment was performed before inoculation of wells with HMs and Abs.

Ab-sensitive *S. aureus* NCTC 12973 was used in each experiment batch as a negative control. Incubation was performed in a shaking incubator at 25 °C and 200 rpm for 72 h. OD readings from each well were recorded at time zero, before inoculation of wells with antimicrobials (to monitor OD changes due to antimicrobial agents) and at 6 h intervals. To maintain culture humidity, 2.5 L of water was incubated with the microplates. Plate reading was performed at time zero and 6-hour intervals at 600 nm (FLUOstar^®^ OPTIMA, BMG LABTECH, Auckland, New Zealand). Bacterial resistance was quantified as MIC based on the obtained MIC results at the exponential growth phase, and data were analysed according to the EUCAST ECOFF (epidemiological cut-off) recommendations.

### 4.7. Broth Microdilution

Bacterial isolates from three subsites with different degrees of HM intensity (B17: high, B14: intermediate, B10: low) were analysed to determine MIC towards HMs (Cd, Zn, Hg) and antibiotics (Tc, Cm, Ery, Cb, Amp). The assay was performed on 96-well microplate plates, each containing a 99 µL aliquot of liquid culture (cell density adjusted to 5 × 10^5^ mL^−1^) and a 1 µL aliquot of the Abs or HM stocks. Each treatment condition had three technical replicates. Controls included a positive bacteria growth control comprised of a mixture of 1 µL DMSO and 99 µL of fresh broth media, a negative control (sterile media without bacterial cells), and media inoculated with *S. aureus* NCTS 12973 (HM/Ab sensitive strain) as a quality control. *S. aureus* NCTS 12973 MIC test values were compared with the EUCAST (European Committee on Antimicrobial Susceptibility Testing) databases. Plates were incubated in a shaking incubator at 25 °C and 200 rpm for 72 h. The time-zero plate reading was performed at 600 nm before bacteria inoculation.

### 4.8. Bacterial DNA Extraction and Cd Gene Amplification

Total bacterial genomic DNA was extracted from 2 mL of overnight R2A broth cultures of 320 Cd-resistant bacterial isolates (40 per soil sample) using the boiling method [60]. Extracted DNA was transferred to 1.5 mL polypropylene microfuge tubes and stored at −20 °C. The quality of the extracted DNAs was quantified using dsDNA spectrophotometry, and A260/230 nm ratios were determined to detect extract quality.

Two of the most common genes encoding Cd resistance in bacterial isolates, *cadA* and *czcA*, were amplified using the PCR primers and conditions previously described [24,26]. A total of 320 Cd-resistant bacterial isolates (40 per soil sample) were assayed for *cadA* and *czcA*. The *czcA* gene encodes an RND family of efflux pumps involved in bacterial resistance to Cd^2+^, Zn^2+^, and Co^2+^ [61]. The *cadA* gene encodes efflux pumps involved in expelling Cd^2+^ from bacterial cells [62,63]. All PCR products were visualised following electrophoresis at 100 V for 40 min on 2% agarose gels.

### 4.9. Genetic Mobility of Cd Resistance by Conjugation

An overnight broth culture of a streptomycin-resistant (SmR) and Cd-sensitive *P. aeruginosa* ICMP 6286 (International Collection of Microorganisms from Plants (ICMP), Manaaki Whenua/Landcare Research, New Zealand) (denoted MUW001) was used as a recipient strain [64]. MUW001 was used in conjugation assays with donor bacterial isolates as previously reported [12] to investigate the mobility of *czcA* or *cadA* from these isolates.

Donor strains tested for CdR gene transfer were those bacterial isolates carrying *czcA* or *cadA* genes as determined by PCR. Fresh overnight colonies of selected bacterial isolates were patched using sterile wooden toothpicks onto 2% nutrient agar plates concurrent with the preparation of recipient cell cultures. Plates were incubated at 37 °C for 24 h. Squares of sterile velvet cloth were used to print the donor cells onto the recipient strain inoculated on brain heart infusion agar plates containing 100 µg mL^−1^ of streptomycin (Sm) and 1 mM CdCl_2_. The sensitivity of the donor bacterial strains to Sm at 100 µg mL^−1^ was determined prior to strain mating. A Sm-sensitive *P*. *aeruginosa* strain and a bacterial isolate without either *czcA* or *cadA* were used as negative controls. Plates containing mated strains were incubated at 37 °C for 24 h and the growth of transconjugant patches was scored quantitatively.

Transconjugants were tested for resistance to Cd, Zn, or Hg by culturing on nutrient agar plates containing 1 mM of Cd, 5 mM of Zn, or 0.1 mM of Hg, and to Ab by broth microdilution assay using Tc, Cm, Ery, Cb, and Amp. The isolates carrying the mobilisable *cadA* and *czcA* genes were identified by 16S rDNA sequencing using unlabelled 63F and 1087R primers, and the sequencing data were compared to the NCBI nucleotide database using blastn.

### 4.10. Terminal Restriction Fragment Length Polymorphism (TRFLP)

Total genomic DNA of soil samples was extracted and subjected to quality controls and quantification, then TRFLP analysis as described previously [12], and for 16S rDNA sequencing as previously reported [13]. TRFLP data analysis was performed with GeneMapper^®^ software v.4.1 for peak analysis and PRIMER v.7 (Plymouth Marine Laboratory, Plymouth, UK) for NMDS analysis of the relative abundance of T-RFs as a proportion of a total peak height of all the T-RFs in that profile to provide a comprehensive fingerprint from metagenomic samples [65].

### 4.11. S rDNA Sequencing and Bioinformatics

To investigate bacterial community diversity of soil samples, 16S rDNA sequencing was performed. Total genomic DNA samples from soil samples with low (B10), intermediate (B14), and high (B17) HM concentrations were sent to the Massey Genome Service (NZ Genomics Ltd., Massey University, Palmerston North, New Zealand). Illumina 16S V3–V4 rRNA library preparation and Illumina MiSeq 16S rDNA sequencing were performed on these samples. The quality control for 16S rDNA sequencing and the subsequent analysis was achieved with SolexaQA version++ [66] and QIIME 1 [67] software tools to generate a comprehensive taxonomic overview of the soil samples’ microbial communities.

Alpha diversity metrics were calculated for the three subsites using the “qiime diversity alpha” command within qiime_2019.1, with four specific diversity metrics being chosen: “observed_otus”, “shannon”, “simpson”, and “chao1”. The values were exported from the appropriate qza artefacts, resulting in a small table of values.

Taxonomic classification was performed within QIIME 2 [8] using the “qiime feature-classifier function “ the “classify sklearn” option, and the gg-13-8-99-515-806-nb-classifier.qza GreenGenes classifier with default parameters and subsequent tabulation for visualisation. The taxonomic abundances were outputted at level 2 (phylum), which included the “root” and “unknown” categories. These refer to sequences that map to the bacterial kingdom and those that are from unknown phyla, respectively. The data were ordered on average abundance across the three sites, and the top 19 categories were identified. All remaining phyla were then aggregated to form the category “other”. The data were scaled to 10,000 units, and the resulting CSV file was uploaded into the Circos table visualiser (https://mk.bcgsc.ca/tableviewer/, accessed on 22 January 2025), the generated output was downloaded, and the configuration files were adjusted in a circos v.0.69.9 conda environment. The final set of 23 colours used was generated using Colorgorical (http://vrl.cs.brown.edu/color, accessed on 22 January 2025).

### 4.12. Statistical Analysis

A three-way ANOVA of bacterial counts from each soil sample and a comparison of mean values using post hoc Bonferroni correction for multiple comparisons was performed. HM concentration was an independent variable, and moisture content, pH, total C and N content, and Olsen *p*-values were covariates. A four-way ANOVA analysis was conducted for resistant bacteria counts of soil samples on plates with HM and antimicrobial additives. HM concentrations of soils and HM or Ab concentrations in media were independent variables. The dependent variable was bacterial CFU, soil moisture content, soil pH, total soil C and N content, and Olsen *p*-values were covariates. Analyses were performed using SPSS version 29.

A three-way ANOVA analysis was conducted for PICT, and a broth microdilution analysis of soil bacteria and concentrations of HM and Ab in microtiter plates were independent variables. The dependent variable was the MIC value.

A three-way ANOVA was carried out for TRFLP analysis to compare the abundance of the T-RFs between samples and soils. HM concentrations were independent variables, and the dependent variable was the number of T-RFs reads.

## 5. Conclusions

The results of the present study revealed the abundance of bacterial isolates resistant to a range of levels of HM and Abs is greater in soils with high HM content compared to the abundance of those bacteria from soil samples with lower levels of HMs. Moreover, we observed a higher occurrence of HGT for the *czcA* resistance gene in comparison to the *cadA* gene. These findings agree with existing evidence on the effect of HM soil content on HMR, AbR, and gene transference. Furthermore, our data shows members of the Proteobacteria phyla predominate within soil sample bacterial communities. Considering genera from *Pseudomonas*, *Achromobacter,* and *Stenotrophomonas* are well-recognised pathogens that could benefit from AbR, further research into the relationship between HMR and AbR is needed. Our findings support the hypothesis that levels of HM resistance respond to direct selection pressure in the presence of high environmental HM content, while soil bacterial AbR may increase through indirect selection by co-resistant or cross-resistant mechanisms. Contamination of agricultural soil can have long-term consequences. This emphasises the need to protect agricultural soil from a wide range of contaminants to avoid the unintended consequence of selection for AbR.

## Figures and Tables

**Table 1:** BRP subsites soil samples physicochemical information.

Subsite	B10	B11	B12	B13	B14	B15	B16	B17
pH	5.90	6.80	5.90	6.70	6.70	6.40	5.50	6.10
Olsen P *	90.0	96.0	135	180	217	228	265	569
Total C (%)	6.00	5.20	6.40	5.70	6.00	5.90	7.00	8.60
Cd *	1.14	1.15	1.83	2.70	3.40	3.80	4.10	7.20
Hg *	0.109	0.076	0.091	0.079	0.103	0.081	0.129	0.125
Zn *	49.0	48.0	60.0	59.0	68.0	73.0	77.0	95.0
Fe *	19,700	13,400	21,000	18,000	19,200	18,300	20,000	17,100
P *	3300	2100	5000	6300	7700	8500	12,300	22,000
Moisture content (%)	30.0	26.0	27.0	27.0	27.0	27.0	27.0	27.0

* mg kg^−1^ of dry soil.

**Table 2:** Proportions of Cd resistant bacterial isolates from BRP soil samples carrying the *czcA* and *cadA* genes, and frequency of gene mobility.

Soil Samples *	Number of Cd Resistant Isolates	Cd Resistance Genes n (%)		Number of Isolates Carrying Cd Resistance Genes with Gene Mobility n (%)	
		*cadA*	*czcA*	*cadA*	*czcA*
BRP B17	40	1 (2.5%)	37 (92.5%)	0	10 (27%)
BRP B16	40	2 (5%)	35 (87.5%)	0	11 (31.4%)
BRP B15	40	1 (2.5%)	34 (85%)	1 (100%)	8 (23.5%)
BRP B14	40	0	25 (62.5%)	0	7 (28%)
BRP B13	40	0	17 (42.5%)	0	6 (35%)
BRP B12	40	0	11 (27.5%)	0	3 (27.3%)
BRP B11	40	0	8 (20%)	0	2 (25%)
BRP B10	40	1 (2.5%)	8 (20%)	1 (100%)	3 (37.5%)
Total	320	5 (1.6%)	175 (54.7%)	2 (40%)	50 (28.6%)

* B10–B17 identify the subsites where soil samples were taken.

**Table 3:** Metrics of alpha diversity at 3 subsites from the Belmont Regional Park airstrip.

Soil Samples	Observed OTUs	Shannon	Simpson	Chao1
BRP B17	3903	10.801	0.999	3904.737
BRP B14	4505	10.826	0.999	4505.947
BRP B10	5443	10.923	0.998	5448.250

## Data Availability

Data are available from https://mro.massey.ac.nz/items/2584c89a-6985-49f1-a1a1-90fab77ad046 (accessed on 22 Jaunary 2025).

## Supplementary Materials

The following supporting information can be downloaded at: https://www.mdpi.com/article/10.3390/antibiotics14020192/s1, Figure S1: Mean ratios of ZnR/total bacterial CFUs, over a range of Zn concentrations, for BRP subsites soil samples.; Figure S2. Mean ratios of HgR/total bacterial CFUs, over a range of Hg concentrations, for BRP subsites soil samples.; Figure S3: NMDS analysis plot of TRFLP relative peak height for BRP soils’ bacterial communities’ data, using the Bray–Curtis similarity index. Table S1: Comparison of NGS of bacterial 16S rDNA gene reads in various taxonomical levels from B10 and B14 soil samples compared to B17 soil from BRP; Table S2: Individual bacterial isolates able to mobilize CdR by conjugation as donor strains identified by 16S rDNA sequencing.
