# Supplementary material for: Antibiotic and Heavy Metal Resistance in Bacteria from Contaminated Agricultural Soil: Insights from a New Zealand Airstrip
Source: Antibiotics (Basel). 2025 Feb 13;14(2):192. doi: 10.3390/antibiotics14020192 (PMC11851424; doi:10.3390/antibiotics14020192)
Supplement: Supplementary file 1 [file antibiotics-14-00192-s001.zip › antibiotics-3403718-supplementary.pdf]

antibiotics

MDPI

Supplementary Material

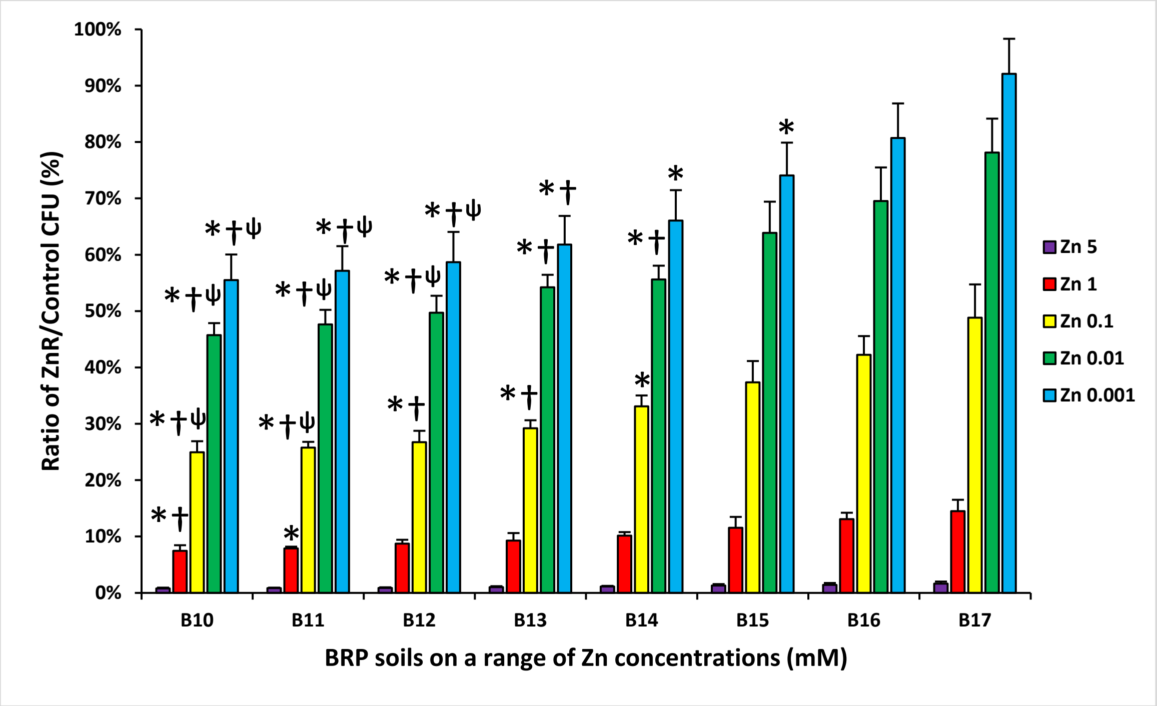

Supplementary Figure S1. Mean ratios of ZnR/total bacterial CFUs, over a range of Zn concentrations, for BRP sub-sites soil samples. *p < 0.05 compared to B17 soil bacteria CFU ratio selected on the same Zn concentration; †p < 0.05 compared to B16 soil bacteria CFU ratio selected on the same Zn concentration; ᵠp < 0.05 compared to B15 soil bacteria CFU ratio selected on the same Zn concentration.

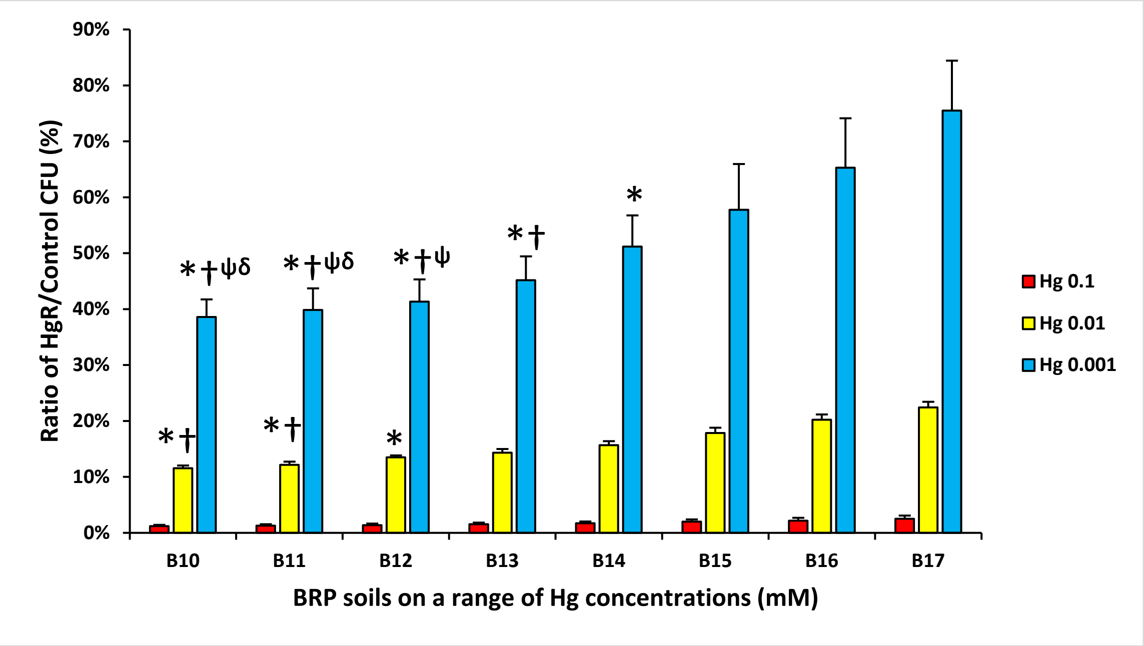

Supplementary Figure S2. Mean ratios of HgR/total bacterial CFUs, over a range of Hg concentrations, for BRP sub-sites soil samples. *$p < 0.05$ compared to B17 soil bacteria CFU ratio selected on the same Hg concentration; †$p < 0.05$ compared to B16 soil bacteria CFU ratio selected on the same Hg concentration; $^{\varphi}$ $p < 0.05$ compared to B15 soil bacteria CFU ratio selected on the same Hg concentration; $^{\delta}$ $p < 0.05$ compared to B14 soil bacteria CFU ratio selected on the same Hg concentration.

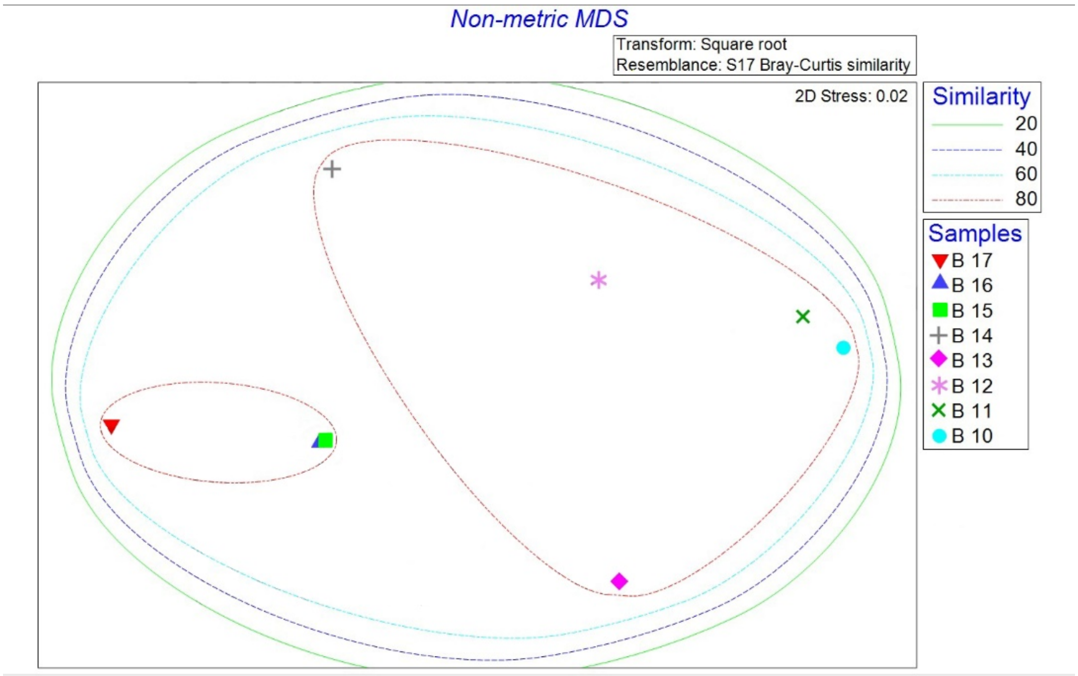

Supplementary Figure S3. NMDS analysis plot of TRFLP relative peak height for BRP soils' bacterial communities' data, using the Bray-Curtis similarity index. Significant difference ($p < 0.05$) between the two clusters specified with >80% of similarity.

Table S1. Comparison of NGS of bacterial 16S rDNA gene reads in various taxonomical levels from B10 and B14 soil samples compared to B17 soil from BRP.

**Taxonomy levels**	**B10**	**B14**
Phylum	$p = 0.018$	$p = 0.039$
Class	$p = 0.0015$	$p = 0.031$
Order	$p < 0.001$	$p = 0.0221$
Family	$p < 0.001$	$p = 0.0217$
Genus	$p < 0.001$	$p = 0.0149$
Species	$p < 0.001$	$p = 0.0146$

Table S2. Individual bacterial isolates able to mobilize CdR by conjugation as donor strains identified by 16s rDNA sequencing.

Bacterial isolate ID	Soil Sample	Cd resistance gene	Description	Accession number	Percent identity
MUW239	BRP_B17	*czcA*	*Cupriavidus sp.* strain JS3054 16S rRNA gene, partial sequence.	MH588163.1	99.40%
MUW244	BRP_B17	*czcA*	*Achromobacter xylosoxidans* strain E2 16S ribosomal RNA gene, partial sequence.	MK849863.1	99.20%
MUW252	BRP_B17	*czcA*	*Alcaligenes sp.* 242 16S rRNA gene, partial sequence.	KT461862.1	99.10%
MUW257	BRP_B17	*czcA*	*Stenotrophomonas sp.* strain D1 16S rRNA gene, partial sequence.	MH814356.1	99.12%
MUW260	BRP_B17	*czcA*	*Pseudomonas protegens* strain Exi5-13 16S rRNA gene, partial sequence.	MK235212.1	99.10%
MUW268	BRP_B17	*czcA*	*Xanthomonas sp.* PG15 16S rRNA gene, partial sequence.	KU350604.1	90.22%
MUW269	BRP_B17	*czcA*	*Stenotrophomonas maltophilia* partial 16S rRNA gene, isolate R5_A9_IIIA.	LR215089.1	98.52%
MUW290	BRP_B17	*czcA*	*Pseudomonas sp.* strain ICMP 22295 16S rRNA gene, partial sequence.	MH392636.1	99.50%
MUW306	BRP_B17	*czcA*	*Serratia proteamaculans* strain P4_BA1R 16S rRNA gene, partial sequence.	MK883049.1	99.22%
MUW307	BRP_B17	*czcA*	*Chryseobacterium nakagawai* strain NCTC13529 genome assembly, chromosome: 1.	LR1234386.1	82.50%
MUW345	BRP_B16	*czcA*	*Stenotrophomonas maltophilia* strain DGN5 16S rRNA gene, partial sequence.	MK764970.1	91.53%
MUW348	BRP_B16	*czcA*	*Pseudomonas geniculata* strain IARI-HHS1-19 16S rRNA gene, partial sequence.	KF054771.1	95.33%
MUW349	BRP_B16	*czcA*	*Achromobacter pestifer* strain LMG 3431 16S rRNA, partial sequence.	NR_152016.1	99.21%
MUW350	BRP_B16	*czcA*	*Rhodococcus sp.* strain OB0511_247-1 16S rRNA gene, partial sequence.	KY020332.1	99.90%
MUW355	BRP_B16	*czcA*	*Achromobacter sp.* JW31.5a partial 16S rRNA gene, strain JW31.5a.	FN556572.1	99.11%
MUW356	BRP_B15	*czcA*	*Pseudomonas palleroniana* strain IHB B 7133 16S rRNA gene, partial sequence.	KJ767328.1	99.80%

MUW358	BRP_B15	*czcA*	*Serratia sp.* SP19E 16S rRNA gene, partial sequence.	KP126635.1	99.61%
MUW365	BRP_B15	*czcA*	*Achromobacter sp.* strain FW305-C-28 16S rRNA gene, partial sequence.	MK402967.2	99.12%
MUW367	BRP_B15	*czcA*	*Serratia sp.* A2 16S rRNA gene, partial sequence.	EU287454.1	99.71%
MUW376	BRP_B14	*czcA*	*Stenotrophomonas maltophilia* clone B2.18.23 16S rRNA gene, partial sequence.	AY837730.1	99.80%
MUW377	BRP_B14	*czcA*	Bacterium strain YCR3A-3 16S rRNA gene, partial sequence.	MF143454.1	99.80%
MUW379	BRP_B14	*czcA*	Bacterium strain BS1294 16S rRNA gene, partial sequence.	MK824482.1	99.51%
MUW388	BRP_B14	*czcA*	*Chryseobacterium sp.* strain DEM Bc1 16S rRNA gene, partial sequence.	MG893574.1	99.40%
MUW390	BRP_B13	*czcA*	*Pseudomonas fluorescens* strain B16-231 16S rRNA gene, partial sequence.	MK072682.1	99.70%
MUW391	BRP_B13	*czcA*	*Pseudomonas palleroniana* strain IHB B 7133 16S rRNA gene, partial sequence.	KJ767328.1	99.80%
MUW393	BRP_B13	*czcA*	*Pseudomonas palleroniana* strain APC14 16S rRNA gene, partial sequence.	KX528176.1	97.97%
MUW394	BRP_B12	*czcA*	*Chryseobacterium sp.* strain E2-18 16S rRNA gene, partial sequence.	KY476499.1	99.70%
MUW395	BRP_B12	*czcA*	*Variovorax boronicumulans* strain E2B5 16S rRNA gene, partial sequence.	KX881472.1	93.09%
MUW396	BRP_B11	*czcA*	*Achromobacter sp.* strain HBUM200336 16S rRNA gene, partial sequence.	KY945518.1	99.28%
MUW401	BRP_B10	*cadA*	*Microbacterium sp.* strain PHIL_400ppmZn_ML16 16S rRNA gene, partial sequence.	MK652511.1	99.59%
